# Latent profiles of psychological empowerment in Chinese police officers: links to emotional support and work well-being

**DOI:** 10.3389/fpsyg.2025.1694664

**Published:** 2026-01-21

**Authors:** Yuan Tian, Zhe Jia, Na Li, Yunpeng Wu, Jue Deng, Ya’nan Tang, Jinshu Huang, Xizheng Xu

**Affiliations:** 1 Department of Criminal Investigation, Hunan Police Academy, Changsha, China; 2 Scientific Research Division, Qilu Medical University, Zibo, China; 3 School of Public Health, Qilu Medical University, Zibo, China; 4 School of Nursing, Qilu Medical University, Zibo, China; 5 Hubei Preschool Teachers College, Wuhan, China; 6 School of Teacher Education, Dezhou University, Dezhou, China; 7 Fujian Police College, Fuzhou, Fujian, China

**Keywords:** emotional support, latent profile analysis, police officers, psychological empowerment, work well-being

## Abstract

**Introduction:**

Psychological empowerment is a critical factor for employee work well-being, particularly within high-stress professions such as policing. However, experiences of empowerment among individuals are not uniform. This study aims to identify distinct profiles of psychological empowerment among police officers and to examine their associations with perceived coworker support and work well-being.

**Methods:**

A person-centered approach was adopted. Data were collected from 505 Chinese police officers. Latent Profile Analysis (LPA) was employed to identify subgroups based on their psychological empowerment patterns.

**Results:**

The analysis revealed two distinct profiles: a “Globally Disempowered” profile and a “Globally Empowered” profile. Perceived emotional support from coworkers was a significant predictor of profile membership, where higher levels of support increased the likelihood of belonging to the empowered group. Furthermore, officers in the high empowerment profile reported significantly greater work well-being compared to those in the low empowerment profile.

**Discussion:**

The findings underscore the heterogeneity in psychological empowerment experiences within the policing context. They emphasize the pivotal role of fostering emotional peer support as a means to enhance officers’ psychological empowerment and, consequently, their work well-being. Practical implications for organizational interventions are discussed.

## 1 Introduction

Police work is marked by sustained psychological pressure, rigid organizational structures, and frequent exposure to volatile situations, making individual psychological resources essential for maintaining functional resilience (Violanti et al., 2017). Among these resources, psychological empowerment—defined by meaning, competence, self-determination, and impact (Spreitzer, 1995)—has been shown to enhance persistence, judgment quality, and adaptive functioning in high-demand settings. Research in policing consistently documents empowerment’s associations with reduced burnout, stronger identification, and more effective discretionary decision-making (Laschinger et al., 2004; Tummers et al., 2015; Kim and Beehr, 2018).

Despite these established benefits, existing studies overwhelmingly treat psychological empowerment as an aggregate variable to examine its relationships with other variables such as work engagement and innovative behavior (Llorente-Alonso et al., 2024; Seibert et al., 2011), relying on variable-centered methods that conceal the possibility that officers internalize the four dimensions in qualitatively different ways. Given policing’s hierarchical culture, uneven autonomy across roles, and differential exposure to critical incidents, it is plausible—yet empirically untested—that officers form distinct empowerment configurations. Such configurations may reflect different pathways through which job resources translate into motivational and well-being outcomes. Addressing this limitation requires a person-centered perspective capable of mapping the heterogeneity embedded within empowerment experiences.

Building on this idea, scholars increasingly argue that empowerment should be conceptualized not as a single linear construct but as a pattern of co-occurring perceptions (Gong et al., 2021). Distinct profiles—such as high competence but low impact or high meaning but limited autonomy—may lead to different motivational climates and well-being consequences. These insights highlight the need for research that moves beyond mean-level empowerment and considers how individual officers combine and interpret the four empowerment dimensions.

Alongside empowerment, peer emotional support is a central interpersonal resource in policing. Officers rely heavily on peers during operational stress, making peer support a likely contributor to empowerment development. Yet prior research treats peer support as a uniform booster of empowerment, overlooking the possibility that its effects vary depending on an officer’s underlying empowerment pattern. For example, emotional validation may strengthen perceived impact for some officers but reinforce competence for others. Understanding these differentiated pathways requires examining peer support as a predictor of latent empowerment profiles rather than assuming homogeneous effects.

Finally, although empowerment is known to promote work well-being—including satisfaction, engagement, and emotional vitality—the literature has not examined whether different empowerment profiles confer different levels of well-being. Theoretical frameworks such as resource–demand fit suggest that some patterns (e.g., high meaning combined with limited perceived control) may create motivational tension, while more balanced empowerment profiles may serve as robust protective resources. Clarifying these distinctions is particularly consequential in policing, where well-being deficits can impair performance and public safety.

To address these critical gaps, the present study employs a person-centered paradigm through Latent Profile Analysis (LPA) to examine psychological empowerment among frontline police officers. Guided by the Job Demands-Resources model, this research specifically aims to: (a) identify distinct latent profiles based on the four empowerment dimensions; (b) test whether peer emotional support differentially predicts membership in these profiles; and (c) assess how these profiles are uniquely associated with work well-being. By integrating a person-centered framework with the Job Demands–Resources model, this study provides a more nuanced understanding of empowerment in policing and offers insight into how interpersonal resources shape officers’ psychological functioning.

### 1.1 Psychological empowerment

Psychological empowerment refers to a motivational state in which employees perceive their work as meaningful, feel capable of performing required tasks, experience autonomy in initiating action, and believe their efforts influence relevant outcomes (Conger and Kanungo, 1988; Spreitzer, 1995). These four components represent distinct yet interrelated evaluative processes through which individuals interpret their work environment. The multidimensional structure is theoretically grounded in SDT, social cognitive theory, and the job characteristics model, each emphasizing different mechanisms through which empowerment supports internal motivation and adaptive behavior (Bandura, 1986).

Empirical findings show that empowerment promotes a wide range of positive outcomes—higher performance, engagement, creativity, and lower burnout (Seibert et al., 2011). Within policing, empowerment is particularly relevant: officers must frequently make rapid judgments, manage emotional strain, and operate with varying levels of discretion. Studies indicate that empowered officers exhibit stronger emotion regulation, less exhaustion, and greater organizational commitment (Laschinger et al., 2012; Tummers and den Dulk, 2013).

Yet this body of research typically treats empowerment as a single aggregate variable (Llorente-Alonso et al., 2024; Seibert et al., 2011), overlooking whether officers differ in how strongly they endorse each dimension (Gong et al., 2021). Recent research has begun to address this limitation by employing person-centered approaches, such as latent profile analysis, to identify distinct subgroups of employees based on their configuration of empowerment dimensions. Emerging person-centered studies in other occupational contexts identify distinct empowerment patterns that correspond to different motivational orientations and well-being profiles (Gillet et al., 2023; Saward et al., 2024). Whether similar patterns exist in policing—an environment where autonomy and perceived impact vary substantially—remains unknown. Identifying these potential subgroups is essential for understanding how empowerment operates under conditions of structural constraint.

Based on this rationale, we hypothesize that distinct subpopulations of officers exist, characterized by unique configurations of the four empowerment dimensions. Formally stated:

*H1*: Distinct latent profiles of psychological empowerment will emerge among police officers, reflecting meaningful heterogeneity in how the dimensions of meaning, competence, self-determination, and impact combine.

### 1.2 Peer emotional support and its role in empowerment

Peer emotional support encompasses the empathy, validation, and affective reassurance exchanged among colleagues. In the high-stress, high-interdependence context of policing, where peers share duties, risks, and unique occupational stressors (Agarwal et al., 2020; Gullon-Scott and Longstaff, 2024), such support constitutes a critical and culturally embedded interpersonal resource (Brough and Pears, 2004). The Job Demands-Resources (JD-R) model explicitly conceptualizes peer support as a core job resource that buffers the deleterious effects of job demands and actively fuels motivational processes (Bakker and Demerouti, 2007). Complementarily, Self-Determination Theory (SDT) posits that emotionally supportive interactions satisfy the fundamental psychological need for relatedness, and by fostering a climate of trust, can indirectly bolster feelings of autonomy and competence, thereby deepening meaningful engagement in one’s role (Ryan and Deci, 2000).

Empirical evidence across organizational sectors robustly confirms that peer support is positively associated with increased psychological empowerment, job satisfaction, and work engagement, while concurrently reducing burnout and emotional exhaustion (Fong and Snape, 2015; Chiang and Hsieh, 2012). Research specific to public safety occupations further links strong peer support networks to lower trauma-related symptomatology and improved overall emotional functioning (Maguen et al., 2017).

However, a critical gap remains in how this relationship is understood. Prior research predominantly assumes a uniform, linear relationship whereby peer support generically “boosts” overall empowerment levels. This assumption overlooks the complex, person-specific dynamics that may operate within the social ecology of policing. Factors such as unit culture, officer tenure, task-specific autonomy, and pre-existing psychological dispositions likely shape how peer support is interpreted and internalized. For instance, emotional validation from peers might primarily reinforce one officer’s sense of competence, amplify another’s perceived impact, or chiefly address a third’s need for relatedness.

Therefore, moving beyond a uniform view requires a person-centered approach. It is not merely whether support increases empowerment, but whether it influences an officer’s likelihood of belonging to one distinct psychological profile over another. We posit that peer emotional support functions as a differential catalyst, making it more probable for an officer to develop and sustain a harmonious, high-empowerment profile by providing a “safe space” that enhances psychological safety, helps reframe challenges, and reinforces the meaningfulness of work (Fong and Snape, 2015). Consequently, we propose the following hypothesis:

*H2*: Higher levels of perceived peer emotional support will increase the likelihood of police officers belonging to more adaptive, higher-empowerment profiles.

### 1.3 Psychological empowerment and work well-being

Work well-being reflects employees’ integrated cognitive and affective evaluations of their work experiences, encompassing core dimensions such as job satisfaction, work engagement, and emotional vitality (Warr, 2007). A robust body of theoretical and empirical literature consistently identifies psychological empowerment as a key antecedent of well-being. The mechanism is well-understood: empowered employees experience a greater sense of control, purpose, and efficacy in their tasks, which mitigates psychological strain and cultivates positive affect (Ryan and Deci, 2000; Spreitzer et al., 2005). Meta-analytic and longitudinal studies further substantiate this positive linkage across professions (Lee et al., 2022; Laschinger et al., 2004).

In the high-stress context of policing, where chronic operational pressure is endemic, empowerment is similarly associated with critical outcomes such as lower burnout, more adaptive coping strategies, and enhanced emotional functioning. However, mirroring a fundamental limitation within empowerment research itself, investigations into these well-being outcomes have predominantly treated officers as a homogeneous group, analyzing relationships at the aggregate, variable-centered level. A pivotal, unanswered question therefore emerges: Are all configurations of psychological empowerment equally beneficial for well-being? Theoretical reasoning strongly suggests that the internal structure or pattern of empowerment perceptions may be paramount. Drawing on the concept of resource caravanization within conservation of resources theory, a high and balanced profile across all four dimensions likely represents a synergistic, self-reinforcing cluster of psychological resources that maximizes well-being (Hobfoll, 2002). Conversely, imbalanced or mismatched profiles could be internally dissonant and thus less protective. For instance, a profile marked by high meaning but low autonomy or impact—potentially termed “frustrated idealists”—might experience significant motivational tension and emotional exhaustion despite a moderate overall empowerment score, as the unmet need for control frustrates the drive for purpose (Zhang et al., 2023).

Understanding these nuanced distinctions is crucial for moving beyond generic interventions. The well-being returns on organizational efforts to foster empowerment may depend critically on promoting specific, harmonious configurations rather than merely elevating scores on isolated dimensions. Based on this rationale, we hypothesize a differential relationship between empowerment profiles and well-being:

*H3*: Work well-being will differ significantly across the identified psychological empowerment profiles, with more harmonious and higher-level profiles being associated with superior well-being outcomes compared to imbalanced or lower-level profiles.

## 2 Method

### 2.1 Participants

A total of 505 frontline police officers from multiple provincial public security departments in mainland China participated in the study. Data were collected through an anonymous online survey administered via a secure survey platform. To reduce duplicate submissions, IP address restrictions and device verification procedures were implemented. Prior to participation, respondents were informed of the voluntary nature of the study, anonymity protections, and the exclusive use of aggregated data for research purposes.

The final sample included 264 male officers (52.3%) and 241 female officers (47.7%). Participants ranged in age from 20 to 52 years (*M* = 32.21, SD = 4.36). All respondents were active-duty officers engaged in operational or field-oriented duties at the time of data collection. The overall response rate was 84.17%, indicating strong engagement and adequate representativeness for frontline policing populations.

### 2.2 Data analysis procedure

Analyses were conducted in three stages. First, Latent Profile Analysis (LPA) was performed to identify distinct subgroups of officers based on their multidimensional psychological empowerment scores. This person-centered method enables the detection of naturally occurring configurations across the four empowerment dimensions and overcomes the limitations associated with variable-centered assumptions of homogeneity. Second, the R3STEP procedure (Robust Three-Step Approach; Asparouhov and Muthén, 2014a) was applied to examine the effects of external covariates—including emotional support, gender, age, and work experience—on class membership. This approach accounts for measurement error in class assignment and yields unbiased estimates of covariate effects. Third, differences in work well-being across the latent profiles were examined using the BCH method (Bolck–Croon–Hagenaars; Asparouhov and Muthén, 2014b). The BCH technique is considered the gold standard for distal outcome comparisons because it preserves latent class separation and adjusts for classification uncertainty.

All analyses were conducted in Mplus 8.3, using maximum likelihood estimation with robust standard errors (MLR). Model selection was guided by fit indices (AIC, BIC, SSA-BIC), entropy, likelihood ratio tests (LMR, BLRT), and substantive interpretability.

### 2.3 Measures

#### 2.3.1 Psychological empowerment

Psychological empowerment was assessed using the Chinese adaptation of Spreitzer’s (1995) scale (Li et al., 2006). The scale comprises four dimensions—meaning, competence, self-determination, and impact—each measured with three items on a 6-point Likert scale (1 = strongly disagree, 6 = strongly agree). A sample item is: “I can decide on my own how to go about doing my work.” Prior studies have demonstrated strong reliability and construct validity in Chinese organizational environments. In the present sample, the scale showed excellent internal consistency (*α* = 0.97).

#### 2.3.2 Employee well-being

Employee well-being was measured using a 6-item scale developed by Zheng et al. (2015), which captures affective and cognitive evaluations of one’s work experience. Participants indicated agreement using a 6-point Likert scale (1 = strongly disagree, 6 = strongly agree). The scale has shown robust psychometric properties in Chinese occupational contexts. In this study, the internal consistency reliability was high (*α* = 0.93).

#### 2.3.3 Emotional support

Perceived emotional support from coworkers was measured using a 5-item scale adapted from Methot et al. (2015), originally derived from Schaefer et al. (1981). Items were rated on a 6-point Likert scale (1 = strongly disagree, 6 = strongly agree). A sample item is: “My coworkers provide encouragement and emotional support.” A composite score was calculated by averaging the items, with higher scores indicating stronger perceived emotional support. The scale demonstrated excellent internal reliability in this sample (*α* = 0.95).

## 3 Results

### 3.1 Common method Bias

Given that all focal variables were self-reported, potential common method bias (CMB) was evaluated. First, a three-wave time-lagged design was employed to reduce common rater and consistency motifs, following recommendations by Podsakoff et al. (2003). Second, Harman’s single-factor test indicated that the first unrotated factor accounted for 26.99% of the variance, below the 40% threshold, suggesting that no single factor dominated the covariance structure. Third, variance inflation factor (VIF) values for all variables were <1.50, well below the recommended cutoff, indicating an absence of multicollinearity and reducing concern that CMB substantially biased the estimates. Overall, these analyses suggest that CMB is unlikely to pose a serious threat to the validity of the findings.

### 3.2 Latent profile analysis of psychological empowerment

Latent Profile Analysis (LPA) was conducted using the four psychological empowerment dimensions—meaning, competence, self-determination, and impact—as continuous indicators. Models specifying one through four latent profiles were estimated and compared using established fit criteria, including AIC, BIC, adjusted BIC, entropy, and the Lo–Mendell–Rubin adjusted likelihood ratio test (LMR-LRT).

**Figure 1:**
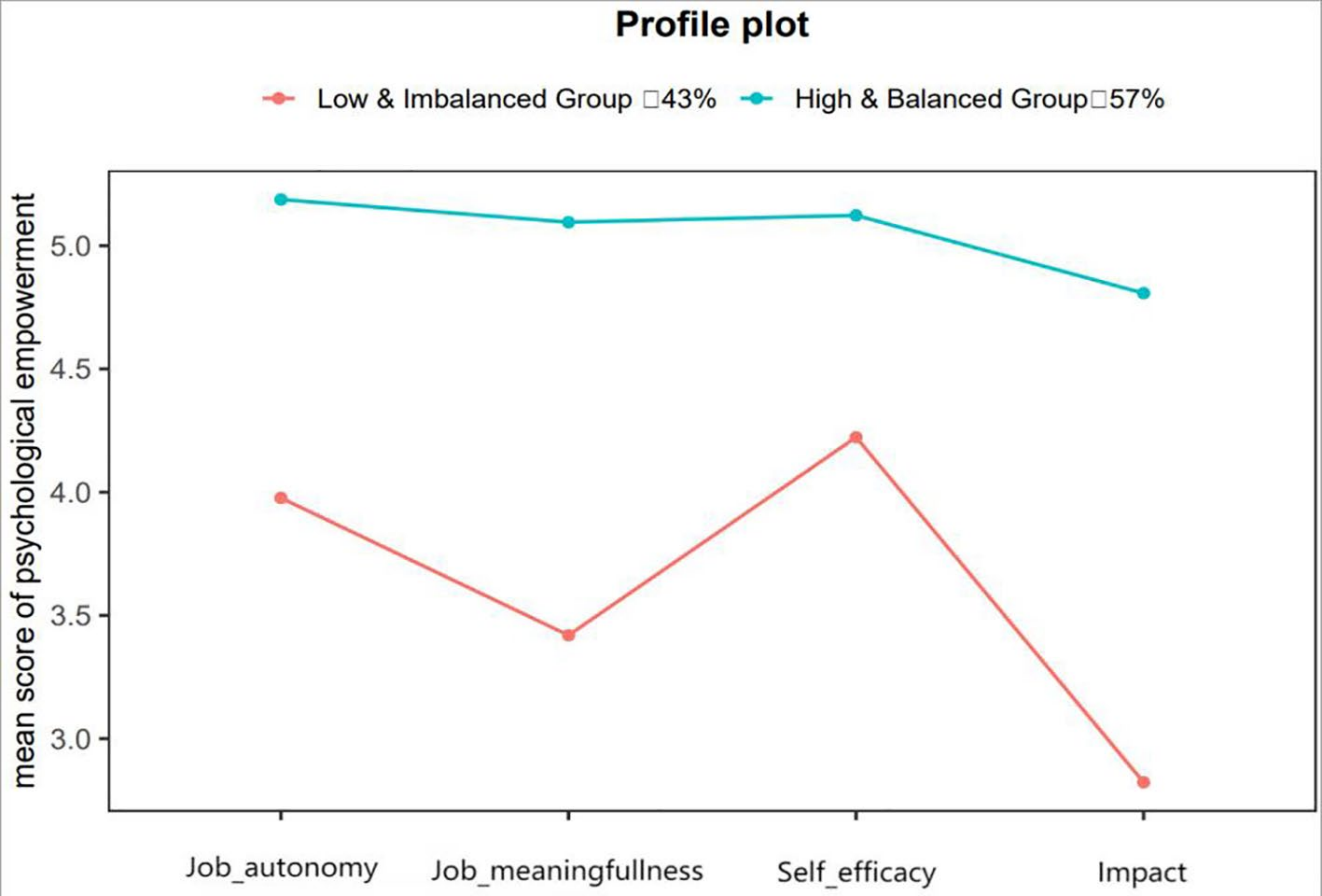
Profile plot of psychological empowerment.

As shown in Table 1, the two-profile model demonstrated the optimal balance between statistical fit and theoretical interpretability. It showed relatively lower AIC/BIC values, an entropy of 0.80, and a significant LMR-LRT (*p* < 0.001), indicating that adding a second profile significantly improved model fit. Additional profiles did not yield meaningful interpretive advantages and showed declining fit improvements. Inspection of standardized mean scores (Figure 1) supported clear differentiation between the two profiles: Profile 1: Low and Imbalanced Psychological Empowerment (C1; 43%; *n* = 104) Characterized by consistently low levels across all empowerment dimensions and marked unevenness among indicators. Profile 2: High and Balanced Psychological Empowerment (C2; 57%; *n* = 139) Defined by uniformly high and well-aligned scores across meaning, competence, self-determination, and impact. These patterns indicate that police officers differ not only in magnitude but in the configuration of empowerment, underscoring the value of a person-centered approach.

**Table 1:** Fit indices for latent profile models (1–4 classes).

Models	LL	fp	AIC	BIC	aBIC	Entropy	aLMR_*p*	Group proportions
1 profile	−1349.40	14	2726.81	2775.71	2731.34	NA	NA	1
**2 profiles**	**−1219.46**	**23**	**2484.92**	**2565.26**	**2492.35**	**0.80**	**<0.001**	**0.43/0.57**
3 profiles	−1260.76	36	2593.53	2719.28	2605.17	0.94	0.98	0.26/0.28/0.46
4 profiles	−1255.84	47	2605.69	2769.86	2620.88	0.90	0.54	0.21/0.41/0.16/0.22

LL: Model log-likelihood value; #fp: Number of free parameters; AIC: Akaike Information Criterion; BIC: Bayesian Information Criterion; aBIC: Sample Size-Adjusted Bayesian Information Criterion; Entropy: A measure of classification accuracy for latent class assignment, ranging from 0 to 1. Higher values indicate greater classification precision. When Entropy ≥0.8, the accuracy of latent class assignment exceeds 90%. aLMR, Adjusted Lo–Mendell–Rubin likelihood ratio test; BLRT, bootstrapped likelihood ratio test. The bolded values correspond to the optimal latent profile solution.

### 3.3 Effects of emotional support on profile membership

To examine predictors of latent profile membership while accounting for classification uncertainty, a weighted multinomial logistic regression was performed using posterior probabilities as case weights. This approach aligns with three-step LPA procedures and reduces bias in estimating covariate effects.

Results showed that emotional support from colleagues was the strongest predictor of profile membership. Higher emotional support significantly increased the likelihood of belonging to the high/balanced empowerment profile relative to the low/imbalanced profile (OR = 2.93, *p* < 0.001). Substantively, each one-unit increase in emotional support increased the posterior probability of assignment to the high/balanced profile from 50% to approximately 74.6%, reflecting a 24.6-percentage-point gain. Gender differences also emerged: male officers were more likely to belong to the high/balanced profile than the low/imbalanced group. Results for all predictors are presented in Table 2.

**Table 2:** Multinomial logistic regression predicting profile membership.

Predictors	Odds ratios (C#2 vs. C#1)	SE
Age	0.95	0.048
Gender	1.68*	0.165
Work year	0.99	0.054
Colleagues’	2.93***	0.951

Odds ratios (OR) are reported for the comparison C#2 vs. C#1, where C#1 is the reference profile. Gender was coded as 0 = female, 1 = male. “Colleagues’” refers to perceived emotional support from coworkers. OR > 1 indicates a higher likelihood of belonging to Profile C#2 relative to C#1. SE, standard error. *p < 0.05, ***p < 0.001.

### 3.4 Profile differences in work well-being

To assess whether empowerment profiles differed in officers’ work well-being, posterior probability–weighted comparisons were conducted in R. Weighted mean differences were evaluated using 5,000 bootstrap replicates to obtain robust confidence intervals.

A significant overall effect emerged (*t* = 297.60, *p* < 0.001), indicating that latent profile membership was strongly associated with work well-being. Officers in the High and Balanced Psychological Empowerment group reported substantially higher well-being (*M* = 4.92) compared with those in the Low and Imbalanced group (*M* = 3.80). The mean difference of 1.12 points was statistically significant, with a 95% confidence interval of (0.88, 1.35), confirming a large and practically meaningful effect. These findings indicate that empowerment configurations exert a consequential influence on officers’ psychological functioning and subjective work experiences.

## 4 Discussion

This study used a person-centered approach to identify heterogeneous configurations of psychological empowerment among Chinese police officers and to examine how emotional support and work well-being differ across these configurations. The findings reveal substantive intra-group variability in empowerment experiences, highlight the predictive value of coworker emotional support, and demonstrate robust associations between empowerment profiles and well-being. This pattern of results makes two key theoretical contributions. First, it fundamentally challenges the variable-centered paradigm by empirically demonstrating that psychological empowerment is not a homogeneous construct but manifests as qualitatively distinct profiles (e.g., “Globally Empowered” vs. “Disempowered”) within a uniform occupational setting, thereby reconceptualizing it as a heterogeneous configuration of cognitions. Second, it extends the Job Demands-Resources (JD-R) model by revealing that a key interpersonal resource—peer emotional support—functions not merely as a direct buffer, but as a differential catalyst that predicts membership in more adaptive motivational profiles, refining our understanding of how resources operate in high-stress environments. Together, these contributions advance a more differentiated theoretical understanding of empowerment in policing and provide actionable implications for organizational practice, which we elaborate below.

### 4.1 Heterogeneous profiles of psychological empowerment: a bifurcated reality

Latent Profile Analysis revealed two distinct psychological empowerment profiles among police officers: a “Globally Disempowered” profile and a “Globally Empowered” profile. This finding provides direct evidence that officers differ fundamentally not merely in the degree, but in the overall configuration of their motivational perceptions, challenging the long-held variable-centered assumption of empowerment as a homogeneous construct (Seibert et al., 2011). This bifurcation aligns with a growing body of person-centered research that consistently uncovers non-uniform motivational patterns in organizational settings (Morin et al., 2010; Tóth-Király et al., 2021; Wang and Lee, 2009).

The parsimonious two-profile solution is both statistically robust and theoretically meaningful. First, it suggests that for police officers, psychological empowerment may function as a coherent psychological gestalt, where the four dimensions are experienced in a highly interdependent manner. This internal consistency makes statistically stable, intermediate “mixed” profiles (e.g., high meaning but low impact) substantively rare within this population (Spurk et al., 2020). Second, and more critically, this polarized structure likely mirrors the inherently polarizing nature of police work itself. The profession presents a profound tension between the weighty meaning of the mission and the pervasive structural constraints on autonomy and impact imposed by hierarchy and protocol (Tummers and den Dulk, 2013). This “environmental press” appears to push officers toward one of two dominant psychological adaptations: a generally resourceful and aligned state, or a generally constrained and disconnected state. Consequently, the two-profile model is not a methodological artifact but a valid reflection of a bifurcated workplace reality that demands organizational attention.

Officers in the “Globally Disempowered” profile reported uniformly low levels across all four dimensions. This pattern is indicative of a work experience dominated by a sense of ineffectiveness and constraint, likely stemming from and being reinforced by rigid hierarchical structures, limited operational discretion, and procedural rigidity endemic to traditional police organizations (Van der Doef et al., 2022). In stark contrast, officers in the “Globally Empowered” profile reported high and balanced levels across meaning, competence, self-determination, and impact. This configuration represents an optimal motivational state that is fully compatible with the tenets of Self-Determination Theory (Deci and Ryan, 1985), wherein work environments that satisfy core psychological needs for autonomy, competence, and relatedness foster internalized motivation and psychological flourishing.

By moving beyond analyzing average scores to documenting this qualitative heterogeneity, the present findings powerfully underscore the utility of person-centered methodologies. They reveal latent motivational structures that remain obscured in variable-centered analyses. More importantly, this empirical identification of two distinct subgroups provides a compelling foundation for moving away from generic, one-size-fits-all interventions. It directs organizational leadership toward developing differentiated management practices and support systems that are precisely targeted to address the distinct psychological realities of officers in each profile.

### 4.2 Emotional support as a predictor of empowerment profiles

Emotional support from colleagues significantly predicted empowerment class membership: officers who perceived higher coworker emotional support were markedly more likely to be classified in the High Empowerment group. This extends Job Demands–Resources (JD-R) theory (Bakker and Demerouti, 2007) by demonstrating that emotional support—a core job resource—helps shape officers’ motivational configurations rather than merely buffering strain.

The findings also resonate with Conservation of Resources (COR) theory (Halbesleben, 2006), indicating that interpersonal resources may initiate positive resource cycles. Emotional support can strengthen perceptions of autonomy and competence, reinforce psychological safety, and help officers attribute work challenges to manageable causes rather than systemic constraints (Mossholder et al., 2005). In the high-risk police environment, such relational resources are particularly consequential.

Theoretically, this reinforces the view that empowerment is not solely an individual-level psychological state but also an emergent property influenced by team-level relational climates. Practically, it underscores the organizational value of fostering supportive peer cultures.

### 4.3 Empowerment profiles and differential well-being

Outcomes Work well-being differed significantly across empowerment profiles: officers classified as Highly Empowered reported greater job satisfaction, engagement, and emotional well-being. These results align with research showing that empowerment promotes positive affective and health outcomes and reduces burnout (Laschinger et al., 2009; Lee et al., 2022; Kim and Beehr, 2018).

Self-Determination Theory (Ryan and Deci, 2000) provides a coherent framework for interpreting these results. Psychological empowerment enhances the extent to which tasks satisfy autonomy and competence needs, thereby facilitating sustained motivation and well-being. Importantly, the present findings suggest that empowerment operates along a continuum rather than as a binary condition—incremental increases in meaning, competence, or perceived impact can produce meaningful gains in psychological well-being.

In the policing context, where chronic stress and emotional labor are endemic, empowerment may function as a pivotal protective factor. Strengthening empowerment not only enhances well-being but may also support officers’ resilience and professional functioning.

### 4.4 Practical implications

The identification of distinct empowerment profiles has immediate organizational implications. For officers in the Low Empowerment group, interventions should prioritize enhancing autonomy and perceived impact. This could include participatory decision-making structures, clearer performance feedback mechanisms, and job redesign initiatives to increase role significance (Gagné and Deci, 2005).

Given the predictive role of emotional support, agencies should institutionalize mechanisms that reinforce supportive peer interactions. Potential practices include structured peer-mentoring systems, emotional intelligence training, and regular debriefing sessions that provide psychologically safe spaces for officers to process work-related stress (Halbesleben, 2006; Xanthopoulou et al., 2009).

Furthermore, integrating empowerment principles into leadership development and well-being programs may produce synergistic effects, as empowerment contributes simultaneously to motivational enhancement and emotional resilience (Luthans et al., 2007). A sustained organizational focus on empowerment and social support is likely to reduce turnover intention, enhance performance, and ultimately strengthen public confidence in law enforcement institutions.

### 4.5 Limitations and future directions

Several limitations should be acknowledged. First, although the study incorporated a three-wave design to mitigate common method bias, reliance on self-reported data may still introduce response biases. Future research should integrate multi-source or behavioral indicators of empowerment and well-being. Second, the cross-sectional nature of the latent profile analysis limits inference regarding profile development and stability. Longitudinal person-centered models (e.g., latent transition analysis) would enable examination of how empowerment configurations evolve over time or in response to organizational initiatives. Third, the sample consisted exclusively of Chinese police officers, and results may not generalize to policing systems with different organizational cultures or leadership structures. Comparative cross-cultural research would help clarify contextual moderators of empowerment profiles.

## 5 Conclusion

This study identified two distinct psychological empowerment profiles among Chinese police officers—Low Empowerment and High Empowerment—using a person-centered approach. Emotional support from colleagues significantly increased the likelihood of belonging to the High Empowerment profile, indicating its critical role in shaping officers’ empowerment experiences. Moreover, officers in the High Empowerment group reported substantially higher levels of work well-being than those in the Low Empowerment group. Overall, the findings highlight meaningful heterogeneity in empowerment and underscore the importance of coworker emotional support as a key factor associated with better well-being outcomes in policing contexts.

## Data Availability

The raw data supporting the conclusions of this article will be made available by the authors, without undue reservation.
